# Women’s Experiences Regarding Physical Activity during the Postpartum Period: A Feminist Poststructuralist Study

**DOI:** 10.3390/nursrep13010041

**Published:** 2023-03-10

**Authors:** Neda Akbari-Nassaji, Megan Aston, Jean Hughes, Christine Cassidy, Britney Benoit

**Affiliations:** 1 School of Nursing, Dalhousie University, Halifax, NS B3H 4R2, Canada; 2 Rankin School of Nursing, St. Francis Xavier University, Antigonish, NS B2G 2W5, Canada

**Keywords:** physical activity, postpartum, qualitative research, feminist poststructuralism

## Abstract

Although recovery after birth can be promoted through bodily movement, many women do not engage in regular postpartum physical activity. While research studies have identified some of the reasons behind their decisions, including a lack of time, only a limited number of studies have been carried out to explore how postpartum physical activity is socially and institutionally constructed. Thus, the present study aimed to investigate the experiences of women regarding postpartum physical activity in Nova Scotia. Six postpartum mothers participated in semi-structured, virtual, in-depth interviews. Women’s experiences of postpartum physical activity were examined through a discourse analysis guided by feminist poststructuralism. The following themes were identified: (a) socialization in different ways; (b) social support; (c) mental and emotional health; and (d) being a good role model for their children. The findings indicated that all women perceived postpartum exercise as a positive behavior that can promote mental health, although some postpartum mothers experienced social isolation and a lack of support. Furthermore, social discourses about motherhood caused the personal needs of mothers to be disregarded. The results showed that collaboration among health care providers, mothers, investigators, and community groups is necessary to promote and support mothers’ engagement in postpartum physical activity.

## 1. Introduction

The postpartum period is a crucial transition time, and some women find it difficult to participate in physical activity. In recent decades, numerous studies have shown that physical exercise during and after pregnancy has considerable advantages for women and their babies [1,2]. Regular physical exercise throughout the postpartum period may benefit the mental and physical health of the individual, including through weight control [3]. According to Bean and Lesser [4], women who do not have complications during and/or after childbirth can return to physical activity a few days after delivery or once they feel they are ready [4].

While many women believe that physical activity during the postpartum period can be an appropriate decision, the majority of women do not engage in physical activity after childbirth. For instance, Garshasbi et al. [5], in their quantitative research study involving 200 participants in Iran, found that only 3% of postpartum women participated in physical activity at a moderate level. In this research study, they found that a lack of time and energy were the most significant limiting factors for engaging in postpartum physical activity [5]. The same findings were also reported by other researchers in different geographic areas including Australia, the United Kingdom, and the United States [2,6,7]. Moreover, factors including a lack of confidence and motivation were reported as the other important factors that discouraged women from engaging in postpartum physical activity [2].

Extensive research studies have focused on the benefits of exercise during pregnancy and after childbirth, or on identifying the barriers or enablers of postpartum physical activity in order to explain the reasons behind women’s decisions [2,8,9].

A few researchers have attempted to look at the issue of engaging in physical activity from social perspectives [10,11]. Krik [11] believes that society has a crucial role in affecting how behavior is undertaken by individuals, or in explaining the reason for that behavior [11]. More specifically, a limited number of studies indicate how postpartum physical activity is experienced by women and is influenced by dominant social and cultural practices, attitudes, and norms. In order to promote postpartum physical activity it is necessary to understand the beliefs, values, practices, subjectivities, and relations of power among postpartum women residing in a province of Atlantic Canada that has been predominantly constructed through a Western discourse. Feminist poststructuralism is both a theoretical lens and a research methodology [12,13,14]. It can be used to explore how discourses around physical activity after childbirth may influence individuals’ practices. In addition, it offers a rigorous way of comprehending the experiences and reasons why women choose to do exercise following childbirth. The effectiveness of feminist poststructuralism as a lens to explore beliefs, values, and practices of individuals in the postpartum period can be found in the literature [12,13]. Consequently, this study aimed to learn more about how social and institutional factors shape women’s postpartum experiences with physical activity.

## 2. Materials and Methods

### 2.1. Theoretical Framework

This research study applied feminist poststructuralism as a theory [14]. Feminist poststructuralism offers a lens to understand how experiences are personally, socially, and institutionally constructed, and how different subject positions play roles in forming these experiences [15]. According to Weedon [14], “the principles of feminist poststructuralism can be applied to all discursive practices as a way of analyzing how they are structured, what power relations they produce and reproduce, where there are resistances and where we might look for weak points more open to challenge and transformation” ([14], p. 136). It can provide insight into a wide range of health issues by examining how social, institutional, and political constructs shape power relations between people [13]. Important concepts of feminist poststructuralism are language, meaning, beliefs, values, practices, subjectivity, relations of power, and agency [14,16,17]. For example, agency helps to understand how people decide in different situations through challenging or accepting different discourses or meanings associated with their experiences. How people position themselves in relation to others is considered subjectivity [13]. These concepts are used to deconstruct the social and institutional constructions [17]. Moreover, when certain tenets of feminist poststructuralism are used to deconstruct discourses, constructs, and relations of power, solutions may then be illuminated through a process of reconstruction and examination of the taken for granted [17].

### 2.2. The Researchers

A team of nurses who were experts in maternal and child health care, mental health, and marginalized populations carried out this research study. All team members had experience in applying qualitative research methods and feminist poststructuralism. All team members had experience in academic education and clinical practice.

### 2.3. The Qualitative Research Study and Participants

Women who were in the postpartum period (up to 12 months after giving birth) with firsthand experiences regarding postpartum physical activity were recruited using a purposive sampling strategy. They had to be capable of speaking and comprehending English and be at least 19 years old. They also needed to live in Nova Scotia and have a phone or internet connection. As a recruiting approach, electronic poster advertising was utilized. The recruiting advert was posted on the website mumsns.ca and circulated across the website’s connected social media platforms including Twitter, Instagram, and Facebook. For accessible communication, women were given the investigator’s contact information, including their phone number and email address. Fifty postpartum women responded to this call. As the aim of the research study was to develop a comprehensive understanding of women’s experiences through in-depth interviews, and to demonstrate the effective application of the research methodology, we could only interview a limited number of postpartum women.

### 2.4. Data Collection Method

Semi-structured, in-depth interviews were used to collect the data. Each interview was virtually conducted using a telephone due to the COVID-19 outbreak and restrictions. All participants had the opportunity to choose whether they wanted their interview to take place on the phone or using a video platform such as Zoom. All participants preferred to engage in interviews using a phone. All interviews were audio-tape recorded. Every interview took approximately 45–60 min. Every participant had to complete a consent form and submit information about their demographics, including their race and gender, living situation, location, and the number of children they had. Interviews were started with broad questions, after which we used probes depending on participants’ answers. All interviews were carried out by the first author. During the interview, she introduced herself as a maternal–child nurse who was a Master’s student.

### 2.5. Context of the Research Study

This research was carried out in Nova Scotia, a Canadian province mostly encircled by the Atlantic Ocean. Throughout the year, the climate varies from sunny days in summer to windy and snowy weather in winter [18]. Consequently, In Nova Scotia, many outdoor activities can be limited depending on the weather. All interviews were carried out during the month of April 2021 when schools were still closed, and restaurants were just re-opening in Halifax. People were also just beginning to be vaccinated against COVID-19. Therefore, some of the participants’ experiences were impacted by the social restrictions of COVID-19 regarding staying at home.

### 2.6. Data Storage

To maintain confidentiality, the first author of this paper changed all identifying information of participants. All research materials were saved electronically on a private internal OneDrive.

### 2.7. Data Analysis

Transcribing was started after each interview was finished. The research team members examined the interviews independently. Notes and memos were also utilized to preserve the details of the research study. They were added to interviews after finishing each interview.

Discourse analysis was employed to analyze the data. In this research study, the method of discourse analysis was guided by feminist poststructural discourse analysis, which has been recommended by Aston [12]. This open guidance explains that after identifying the beliefs, values, and practices included in the data, discourses, power relations, agency, and subjectivity should be recognized. Special attention should be paid to social, personal, or institutional discourses, and they can take several forms, such as dichotomizing, dominating, suppressing, or being invisible; nonetheless, the context has a considerable influence on them [12]. Data collection and analysis were carried out concurrently. The beliefs, values, and practices were retrieved from every quote by giving special consideration to the participants’ word selection to maintain the meaning derived from their specific experiences. Following that, the quotations related to social and institutional discourse were analyzed. Relations of power were simultaneously incorporated into understandings of how participants used their subjectivity and agency through different discourses. The research reporting adhered to the COREQ reporting guidelines for qualitative research.

### 2.8. Ethical Considerations

Participants were given a complete explanation regarding the study’s aims, potential risks, and benefits, and they provided consent before the interview began. The mothers had the right to withdraw from the study whenever they wished up to one week after their interview. Given that in a qualitative research study people talk about their feelings and experiences, it might lead to anxiety and distress; thus, if a participant felt anxious or distressed due to interview questions, they were provided with a list of mental health centers they could contact if they wanted to speak with a mental health care professional.

## 3. Results

Six women participated in the study, with a mean age of 34 years; the youngest mother was 20 years old and the oldest mother was 41 years old. One participant was a single parent, while the other participants lived with their husbands. One participant was from the Middle East, while five participants were white and from Canada. In terms of residency, five participants resided in cities or towns, whereas just one lived in a rural region. In terms of physical activity, for all participants the definition of physical activity was varied and ranged from walking to regular running. Resuming these activities was difficult for all participants during the postpartum period. Their babies ranged in age from 4.5 to 10 months, with an average age of 7 months. Four women had other children ranging from one to four more. The siblings ranged in age from 2 to 19 years. All participants had education levels higher than high school, ranging from community college to a Master’s degree. The identities of all participants have been altered to maintain confidentiality. The findings were grouped into the following themes: (a) socialization in different ways; (b) social support; (c) mental and emotional health; and (d) being a good role model for their children.

### 3.1. Socialization in Different Ways

Almost all participants expressed that they valued socializing, yet they also expressed concerns that they had minimal opportunities to socialize. Socialization was valued by participants for a variety of reasons: Some participants liked to participate in postpartum physical activity to deal with feelings of isolation. A few participants liked to participate in postpartum physical activity to ensure the normal health and development of their babies. The other participants liked to engage in postpartum physical activity to improve their mental health.

Postpartum isolation was reported by some participants, and they made an effort to interrupt this feeling by finding particular ways to meet others via physical activity. For example, Anne (participant #3) valued doing physical activity with a friend; thus, she would not feel alone and could get some exercise. She explained during the interview:

*When you have someone else, even if you’re not talking, they’re just jogging behind you or practicing beside you, it just feels like you don’t feel isolated, you feel like you’re part of a bigger group.*

Socialization for a few participants was complex. Some participants wanted to keep the children close to themselves because it gave them a sense of security, yet at the same time they wanted to engage in postpartum physical activity with another person. For Anne (participant #3), being away from her baby bothered her, as she mentioned during the interview:

*I was still very much trying to take care of my baby and figure out what’s the best way to take care of him, what are the best things to do … It’s just very stressful because you have this other person that you need to take care of, and if you don’t take care of them, you feel like they can’t do anything for themselves, so you continuously have to be beside them, you have to be watching them.*

This participant (#3) described her postpartum situation as stressful because it was a new experience with her baby to try to figure out how best to care for him. She felt that she had to ‘be beside’ him and ‘watch’ him ‘every single second’. This made it difficult for her to leave her baby, exercise, and socialize. However, she did. Anne explained during the interview:

*My son, I don’t like being away from him. It bothers me when I’m away from him. That prevents me from being excited or motivated to exercise.*

She feels emotionally responsible for her infant, which decreased her “motivation” to exercise. Her values and beliefs about caring for her infant influenced her decision to exercise. For Anne, being a mother meant that she had to be continuously with her child. Anne believed that her subject position as a mother needed to include constant care. This might be influenced by a mothering discourse that perpetuates the idea that mothers need to be with their babies all the time in the early stages of postpartum. Anne undoubtedly shared this belief. It was this belief that was making her decision to exercise on her own away from her baby difficult.

Other participants were also concerned about their socialization. For example, for Caroline (participant #6), interacting with other people gave her a sense of being healthy and “normal.” She explained that engaging in postpartum physical activity with other individuals created the opportunity for her child to interact with other children. Moreover, mothers could support each other in terms of taking care of their babies, talking about positive ideas, and getting rid of negative thoughts.

*It keeps me grounded, and it keeps me being able to talk with other people, and be able to socialize, and make me feel better to be able to socialize and be normal (Caroline).*

Caroline’s subjectivity as a mother was evident from her notion that she, as a mother, had the responsibility to make sure that the growth and development of her baby was similar to that of other children, and that she felt she was doing her duties correctly, which gave her a sense of comfort.

*Just knowing that you’re normal and feel okay, and that your children interact with other children, or you get to see your baby and other babies and realize that everything’s okay and everything’s normal …… Whenever you’re home, you can’t have access to that, or other people and other things. You just do it yourself as a mom, because you don’t really know if things are really going right.*

Caroline had been diagnosed with an anxiety disorder 15 years previously and, when she experienced isolation and doubt, wondered if this might have contributed to her concerns about what was normal and healthy for herself and her baby. This feeling of isolation was created by her postpartum circumstances of being at home without contact with other people outside of her home. This was partly created by social discourses of postpartum, in which parents are often at home by themselves with a newborn both physically and emotionally, without support from family or friends. This was also exacerbated by COVID-19 restrictions, whereby people were asked to stay home as much as possible. Caroline decided to use her agency and chose to find other parents to spend time with through physical activity, so she could talk with them and feel normal. She chose to not perpetuate the social discourse that expected mothers to be able to figure out how to take care of their newborn ‘naturally’ on their own [19]. Given that stigma towards people with mental health problems is socially constructed, mental health issues continue to be uncomfortable for people to discuss and are thereby kept invisible or silenced [20,21,22]; Rather than telling people that she had mental health issues, Caroline chose to address them by socializing with friends.

### 3.2. Social Support

During the postpartum phase, all participants constructed social support in diverse ways. All participants acknowledged that it would be easier to engage in physical exercise if they had social support, which included both practical and emotional assistance. A few participants defined social support as a person who provides care for the baby or performs household duties. Without such support, it was not easy to participate in postpartum physical exercise. For one participant being away from her child was a major barrier in spite of her husband being home and providing care for the baby. For other participants the presence of other people who provided care was important. Most participants received this assistance from their partners or family members.

Beth (participant #2), who was from a Middle Eastern country, mentioned that she would like to participate in postpartum physical activity but faced a lack of social and family support. This participant said:

*In my country if a woman wants to give birth, she goes to their mother’s home for 40 days. Prepare the food, she does anything. They take care of the other babies. It is completely different when you’re by yourself in another country, there is no family. Maybe people, your friends will help make meal, food, maybe help you, but it’s not ever like your family. So that for me is also a big challenge. This is maybe for immigrants, but for others who are Canadian they will not face these challenges.*

All participants made it evident that they needed social support to engage in postpartum physical activity, but for each participant the source and types of support were different. Emotional, informational, and instrumental (e.g., tangible assistance) are different types of social support [23]. Participants in this research study stated that they needed either emotional or instrumental support, or both, to engage in postpartum physical activity. For Anne (participant #3) and Sara (participant #4), social support meant a person who encouraged them to participate in postpartum physical activity, and they both received this support from their partners. Anne’s husband provided care for the baby when she was engaging in physical activity. In the following quotes, Anne explained how her husband respected her idea against social and institutional messages that “mothers should be all” for their children [24]. This support made it easier for Anne to decide to engage in postpartum physical activity. She explained during the interview:

*It was really like my husband was also supporting me because he knew how much I loved being active. He was really just encouraging me to take some time for myself.*

Sara valued postpartum physical activity and believed that mothers should overcome challenges that prevented them from doing physical activity by getting help and support from their partners, friends, family members, or community. Sara explained during the interview:

*We should try to take care of ourselves too. We should not let things to get so hard on us. If we need a support, if we need a help, we should ask for it. If the partner or family or none of those are available, I’m sure there’s others from the community or there’s so many things that you could get help.*

Caroline believed that women needed family support in order to be able to do postpartum physical activity:

*For me, I needed someone to look after the other things in the house to know that I could go for a walk, or do something.*

However, despite believing that postpartum physical activity is significant, Caroline had limited engagement in it due to the challenges she faced as she explained during the interview:

*I was very depressed and COVID made things really difficult. Whereas I was 40 and had my children, I have some blood pressure issues, so I had a lot of health issues.*

Caroline’s words illustrated again that postpartum women were in need of instrumental and emotional support to engage in physical activity.

### 3.3. Mental and Emotional Health

All participants noted that they liked to participate in postpartum physical activity in order to experience feeling “happy” and having “fun”. For all postpartum mothers, “happy” and “fun” experiences were important because they had positive effects on their mental health. Some mothers also reported that physical activity helped them to reduce their worries and concerns that they experienced as parents during the postpartum period. For example, Caroline (participant #6) was concerned because she could not fulfill her duties as a mother during the exacerbation of her illness. She valued mental well-being and postpartum physical activity because they gave her the energy she needed to do her duties as a mother. These beliefs were probably influenced by institutional health discourses that support the message that postpartum exercise can reduce the signs and symptoms of postpartum depression [1]. Caroline’s mental health and her subject position as becoming a “better mom” may have contributed to her worries and concerns about her parenting role, which may have been influenced by cultural and social expectations of what parents should be like. Caroline thought that she would be a “better mom” if she could support and guide her children better.

*To me, being a better mom is just being able to keep up with all my chores because there’s times with depression that you can’t always keep up with everything.*

While she acknowledged the importance of both physical and mental health, she thought that the exacerbations of her mental illness made her mental health even more important than the physical health. She said:

*Physical still helps my mental but my mental wellbeing is more important than my physical because mentally I need to be there for my children, so I need to put aside what I feel about my body.*

Overall, it appears that Caroline dichotomized health into two distinct categories: mental health and physical health. She placed more importance on mental than physical health. She may have also challenged the link between physical and mental health.

There was some variation in how participants experienced the effects of exercise on their mental health. While some women believed that physical activity had an impact on their general mental health, others expressed that it had an impact on a particular area of mental health (positive emotions). For instance, Ellena (participant #5), indicated that the goal of physical activity was to “enjoy” it, since enjoyment can reduce stress, and low levels of stress can enhance pleasant feelings, which can improve mental health. She also realized that enjoyment allowed her to alleviate the distress which was related to interacting with her child or dealing with the COVID-19 outbreak.

*I think mentally it lifts my spirit. If I am in a wretch or feeling, I don’t know, frustrated with the baby or with the situation, mentally, I always feel better after I go for a walk … it feels physically good to move my body and feel like I’m doing something good for myself (participant #5).*

She also believed that doing physical activity was enjoyable because it was something she could do just for herself. She positioned herself subjectively against the dominant and normative social construction of mothers who are often expected to be everything for their children [24]. Thus, Ellena’s agency was to engage in postpartum physical activity. Similar feelings were reported by other participants, as Sara (participant #4) mentioned:

*There are things that we do for everybody in the family, making the food. Things that it’s good for the family, but there are things that it’s just I’m doing it for myself just to make myself to be happier.*

It was evident that the majority of participants resisted the common responsibilities that are often socially constructed for mothers, such as they should be “all for their children” [24]. Thus, doing something solely for themselves made them “happy”.

### 3.4. Being a Good Role Model for Their Children

One important value for all participants was that their children engage in physical activity. All participants believed that their own behaviors could affect their child’s behavior, and it could start as early as the first 3–12 months after birth. Some participants started to think about their role to promote physical activity in their children beginning in the postpartum period. In the following quote, Mary (participant #1) explained how she could influence her children with her behavior:

*They see what we do, and they want to be just like us. I see them, if I am holding cell phone; my baby tries to take my cell phone and touch it, or TV remote. … if my baby sees me doing physical activity, he’s more likely to copy that when he’s older.*

There was a specific relation of power between postpartum mothers and their babies. The mothers thought the role modeling helped them to teach their children about their beliefs and values regarding physical activity, using a non-hierarchical approach. For example, Anne (participant #3) realized that she had the potential to educate her child about the advantages of physical activity, instead of telling her son what to do directly as she explained during the interview:

*I’m hoping that when he’s a little bit older and he understands being active and exercising. I guess just the relation between physical activity and being a mother is just teaching your child to also be active.*

Anne’s subjectivity as a mother can be seen in her statement “the relation between physical activity and being a mother is just teaching”. This indicates her belief that there are certain social norms to being a mother. More specifically, she is referring to being part of a mothering discourse that perpetuates the belief that mothers are meant to teach their children. In this instance, she believed that she had a responsibility to teach her baby the importance of being active.

The majority of participants were anxious about how their children would be affected by social media or spending time in front of a screen instead of engaging in physical activity. For example, Sara (participant #4) noted that sitting in front of the television and eating junk food was a stream that was inevitable for her children, but she attempted to prevent it. As a result, Sara viewed herself as a mother who had power and control over what she wanted her child to learn.

*If my kids can see me being active, so maybe they get this message that it’s better to be outside when you can and that is good. Do something or bike or do something rather than sitting right in front of the TV and eat. Basically, eat junk in front of the TV and just playing video games. I’m sure that one day it’d happen to my kids, but if I can push that further and further to not happen anytime soon.*

The advantage of parents’ role modelling is that it can help parents reinforce desired behaviors in themselves. For example, mothers must practice physical activity themselves if they want to promote it in their children.

## 4. Discussion

In this research study, we demonstrated how social and institutional discourses shape women’s experiences of postpartum physical activity. The participants in this study liked to engage in postpartum physical activity to improve their mental health, promote physical activity in their children, and overcome their sense of isolation.

For some mothers, engaging in postpartum physical activity improved their mood and well-being [1,25]. Pregnancy, delivery, and the postpartum period are crucial life events, in which alterations in mental and physical health are possible. There is much evidence illustrating the positive relationship between postpartum physical activity and positive mood and well-being [26,27]. Moreover, other investigators in a systematic review found a positive relationship between postpartum physical activity and a decreased rate of depression in this period [8]. Improved mental well-being can also lead to decreased postpartum depression [26]. It seems that the participants in this research study relied on some medical discourses that promoted engagement in postpartum physical activity. These beliefs and values should be supported by health care professionals and educational programs.

Moreover, all participants in this research study reported that they preferred social support while engaging in postpartum physical activity. Participants’ construction of social support illustrated that many mothers felt the need for emotional or instrumental support, or both, to engage in postpartum physical activity. Most mothers constructed instrumental support as an individual who could take care of the babies or perform household chores. The role of social support in promoting postpartum physical activity has been presented in previous literature [2,28]. The findings also illustrated that one non-Canadian mother was not able to receive enough support from immediate family members as they lived in another country. It showed how different beliefs, values, practices, and the structure of the family may affect mothers’ perceived social support regarding postpartum physical activity. It also illustrated that people from different cultures constructed social support and family support differently. Further, it has implications for health care professionals to consider cultural differences and the availability of resources when they want to provide care, support, or consultation for this group of postpartum women. Government officials, health promotion clinics, health care professionals, non-governmental organizations, and women and their families need to work together to explore the best ways to support postpartum women to engage in postpartum physical activity, and identify existing resources to support postpartum mothers to engage in postpartum physical activity. For example, some non-governmental organizations could set up group exercise classes that offer childcare facilities.

Additionally, mothers “are expected to be fulfilled solely by their roles as mothers while ignoring other desires and needs” ([24], p. 22). If mothers do not fulfil these needs, they are considered to be selfish and careless. Therefore, in several societies and families women’s own needs are ignored. All the women in this research study challenged the discourse of the ever-giving mother and the idea that mothers should be everything for their children, as many of them would like to do something solely for themselves. It is recommended that educational workshops be held for postpartum mothers and their families to teach them about the health needs of mothers in the postpartum period.

The present research also found that postpartum women experienced social isolation and tried to deal with their sense of isolation. The mothers discovered that postpartum physical activity assisted them with meeting new people and minimizing feelings of loneliness. This finding was congruent with Liva’s earlier research study [29]. Most participants in that research were on maternity leave, and many reported experiencing social isolation throughout the postpartum period. These feelings resulted from increased parental responsibilities and commitments imposed on new mothers under pressure to adjust their daily routines to meet their infant’s schedule. It is crucial to handle postpartum social isolation, since it may result in additional health problems such as stress, anxiety, and depression. Postpartum women may use tactics, such as postpartum physical exercise, to feel less isolation [30], which participants in the current study aimed to accomplish. However, according to the conclusions of this inquiry, the COVID-19 outbreak exacerbated the issue. Indeed, all participants’ physical activity was influenced by restrictions due to COVID-19. In response, online support groups or physical activity apps may have been effective. These applications may provide the necessary encouragement for mothers to engage in physical activity [31]. However, more research is needed to explore the effect of these new technologies specifically in promoting postpartum physical activity. Health care professionals, researchers, and community groups working with families should collaborate to find the best approaches to promote physical activity in postpartum women. Additionally, all participants did not like to try new places for engaging in physical activity, meet new people, go to the gyms, or public places due to COVID-19 restrictions. Thus, the COVID-19 outbreak influenced participants’ physical activity directly or indirectly. If participants had somebody to take care of their babies, they could engage in physical activity. Again, it illustrated the importance of providing support to mothers to engage in postpartum physical activity as the postpartum period can be isolating for most parents.

The findings showed that all participants expressed a desire to participate in postpartum physical exercise to be role models for their children. In a research study that was carried out by Garriguet et al. [32], their results showed that there was a direct relationship between parents’ physical activity and children’s physical activity. Parents played a crucial role in encouraging and promoting their children’s physical activity [32]. It is important to note that childhood sedentary behavior contributes to health issues, such as obesity, and is exacerbated by prolonged television viewing and frequent computer use. It is also common to eat junk food when watching television [33]. These behaviors create great worries and concerns in families that were reported by participants in the current study. Fostering parental role modeling of healthy behavior is one of the strategies recommended by experts to prevent a sedentary lifestyle and promote physical activity in children [33]. However, more research is needed to explore the effect of postpartum mothers’ role modeling on infants.

### Limitations

This study only included postpartum mothers who were able to speak English, and all of them were well-educated; thus, the experiences of people who spoke other languages and had a different level of education were not discussed. It is recommended that future studies involve different groups of people in terms of race, culture, level of education, and social economic status. Another limitation was the format of the interviews. The COVID-19 outbreak forced all interviews to be conducted over the phone. Therefore, despite the fact that firsthand data were obtained, comprehending the participants’ body language was not possible. It is recommended that for future studies, various formats of interviews, including face-to-face, video calls, and phone are utilized. Additionally, this study was conducted during the COVID-19 pandemic, and this isolation may have affected the participants’ experiences in unique ways. However, research conducted by previous researchers [15] demonstrated that postpartum experiences of new parents during COVID-19 were seen to be exacerbated. For example, isolation was present before COVID-19 but was elevated during the pandemic.

## 5. Conclusions

This study illustrated that participating in postpartum physical activity was influenced by different discourses on mothering and health. For example, contending with the social discourse that mothers are expected to be primary, selfless care takers influenced the way they chose to participate in postpartum physical activity. Discourses on mental health also impacted the way participants spoke about the way physical activity influenced their own mental health. The belief that mothers are teachers was taken up in different ways for participants when they chose to role model physical activity for their infants and children. The meaning of support was different among mothers depending on how they felt about the relationship they had with their baby and other family members. Participation in physical activity happened more often for mothers who received emotional or instrumental support from their partners or immediate family members than mothers who did not have such support. For a few mothers, support referred to a person who could provide care for their babies or do domestic chores. In summary, it was evident that the postpartum period is a challenging time and deciding how to engage in physical activity is influenced by postpartum discourses.

## Data Availability

Data are unavailable due to ethical restrictions.
